# Suicide rates: age-associated trends and their correlates

**DOI:** 10.5249/jivr.v4i2.101

**Published:** 2012-07

**Authors:** Ajit Shah

**Affiliations:** ^a^International School for Communities, Rights and Inclusion, University of Central Lancashire, Preston and Consultant Psychiatrist, West London Mental Health NHS Trust, London, United Kingdom.

## Abstract

**Background:**

Suicide rates traditionally increased with ageing. There is a paucity of studies examining factors associated with age-associated trends in suicide rates.

**Methods:**

The relationship between suicide rates and ageing was examined by ascertaining suicide rates in the seven age-bands 16-24 years to 75+ years from the World Health Organisation for 97 countries. The relationship between socio-economic status, income inequality, healthcare expenditure, child mortality rates and life expectancy and countries with an increase, a decline and no change in suicide rates with ageing was examined using data from the United Nations.

**Results:**

In males and females there was a decline in 5 and 10 countries, an increase in 33 and 37 countries and no change in 59 and 50 countries respectively in suicide rates with ageing. Age-associated trends in suicide rates were significantly associated with socio-economic status (males) or income inequality (females), per capita expenditure in healthcare, the proportion of gross-national domestic product spent on healthcare, child mortality rates and life expectancy.

**Conclusion:**

The current study, of factors associated with age-associated trends in suicide rates, confirmed a previously developed five sequential stage model to explain the relationship between elderly suicide rates and socio-economic status and income inequality, quality and quantity of healthcare services, child mortality rates and life expectancy.

## Introduction

Traditionally, suicide rates increased with ageing in many countries.^1^However, exceptions to this observation are emerging. Data from the World Health Organisation (WHO) data bank in 1995 revealed that female suicide rates did not increase with age in Mauritius, Colombia, Albania and Finland.^1^A recent cross-national study reported that there was a significant increase in suicide rates with increasing age in males and females in 25 and 27 of the 62 studies countries respectively.^2^The same study reported that there was no increase in suicide rates with ageing in males and females in 31 and 29 countries respectively and in small number of countries suicide rates declined with increasing age. However, this study only used one-year (the latest available year) data on suicide rates and is open to bias due to year on year random fluctuations in suicide rates.

Suicide rates for both sexes increased with age in Switzerland^3^and Brazil,^4^ but there were smaller peaks in the younger age-bands. Although, the female suicide rate increased with age in China, there was an additional peak in the younger the age-bands.^5-7^ Male suicide rates were the highest in the age-band 25-29 years in Thailand.^8^In England and Wales, male suicide rate was the highest in the age-band 25-34 years,^9-11^but female suicide rates increased with ageing in England and Wales.^11^In Northern Ireland and Scotland male suicide rates decreased with ageing and peaked in the age-band 25-34 years, and female suicide rates peaked in the age-bands 25-34, 35-44 and 45-54 years.^11^This latter study is one of the few studies to use five-year average of suicide rates to minimize any bia due to year on year random fluctuations in suicide rates.^11^Suicide rates for Australian, New Zealand and white American males increased with age, but suicide rates for females initially increased with age, peaking at menopause, and declined thereafter.^12-14^Suicide rates among non-white Americans,^13,15^Indians,^16,17^Jordanians,^18^Indian immigrants to the United Kingdom^10,19^and some east European countries^20^ declined with increasing age.

Only one cross-national study has formally examined factors associated with age-associated trends in suicide rates.^21^This study reported that life expectancy was increased and child mortality rates were decreased in countries with an increase in suicide rates with age in males. Moreover, income inequality was lower in countries with an increase in suicide rates with age in females.^21^ However, there was no relationship between age-associated trends in suicide rates and socio-economic status measured by gross national domestic product. 

With the emergence of studies from several countries without an increase in suicides rate with ageing or a decline in suicide rates with ageing, a cross-national study examining the relationship between suicide rates and age was undertaken to ascertain the current world status of this relationship. This study used five-year average of suicide rates to minimize bias due to year on year random fluctuations in suicide rates. Additionally, the relationship between age-associated trends in suicide rates and socio-economic status, income inequality, life expectancy and child mortality rates was also examined to identify possible determinants of age-associated trends in suicide rates. The data used in this study is the latest available from the WHO and more recent than that used in an earlier study using only one-year data on suicide rates^2^by the author’s group and has a greater number of countries.

## Methods

**Data on suicide rates**

Data on suicide rates for males and females in the seven age-bands 15-24 years, 25-34 years, 35-44 years, 45-54 years, 55-64 years, 65-74 years and 75+ years were ascertained from the WHO website (http://www.who.int/ whosis/database/mort/ table1.cfm) for all listed countries. For a small number of countries only the raw figures for the number of suicides were available from the WHO website. Suicide rates for such countries were calculated by dividing the number of reported suicides by the population size in the relevant age-band and sex group available on the same website. Data were ascertained for each of the latest five consecutive years and a one-year average suicide rate was calculated for each age and sex bands. The median (range) for the latest of the five year of the suicide rate data was 2005 (1983-2007); the total number of countries with this data was 97.

**Data on potential associated factors**

Data on the gross-national domestic product (GDP), per capita expenditure on healthcare, the proportion of GDP spent on healthcare, life expectancy and child mortality rates (i.e. mortality before the age of five years) were ascertained from the WHO website (www.who.int/countries/en/) for the year 2006. The GDP was used as a measure of socio-economic status. Data on a measure of income inequality (Gini coefficient) was ascertained from The United Nations Development Programme website for each country (http://hdr.undp.org/en/media/HDI_2008_EN_Tables.pdf). The Gini coefficient is derived from an income distribution curve where the x-axis represents the number of households and the y-axis percentage of the total income. Perfect equality is seen when the income is equally distributed across all the households and perfect inequality is seen when only one household has all the income. The area between the line of perfect equality and the actual income distribution is the Gini coefficient and is expressed as a percentage ranging form 0 (perfect equality) to 100% (perfect inequality). The Gini coefficient was used as a measure of income inequality.

**Data analysis**

Each of the seven age-bands 16-24 years, 25-34 years, 35-44 years, 45-54 years, 55-64 years, 65-74 years and 75+ years were coded numerically in the ascending order of 1 to7. Spearman’s correlation coefficient (rho) was used to examine the relationship between the seven age-bands and the suicide rates by correlating the ascending order numerical codes for the seven age-bands with the absolute suicide rate for each age-band. These analysis were conducted for both sexes for each country. This method of analysis has been successfully used to examine age-associated trends in suicide rates.^2,11^Age-related trends in suicide rates were divided into three groups: increase, no change and decrease in suicide rates with increasing age. The relationship between these age-related trends in males and females and the GDP, per capita expenditure on healthcare, the proportion of GDP spent on healthcare, life expectancy, child mortality rates and the Gini coefficient were examined using the Kruskal-Wallis one-way analysis of variance (for differences between the three groups) and the Mann Whitney U Test (for differences between individual groups).

## Results

**Suicide rates and ageing**

The relationship between suicide rates and age in both sexes in different countries is illustrated in Table 1. A significant decrease in suicide rates with ageing was observed for males and females in 5 and 10 countries respectively.

**Table:** Table 1: **The relationship between suicide rates and age**

Country	Males	Females
Albania	NS	NS
Anguilla	NS	NS
Antigue	NS	NS
Argentina	NS	NS
Armenia	rho=+0.96	rho=+1
	P<0.0001	P<0.0001
Aruba	rho=-0.93	NS
	P=0.003	
Australia	NS	NS
Austria	rho=+0.96	rho=+1
	P<0.0001	P<.0001
Azebaijan	rho=+0.96	rho=+0.86
	P<0.0001	P=0.014
Bahamas	NS	NS
Bahrain	rho=-0.78	rho=-0.79
	P=0.041	P=0.035
Belarus	NS	rho=+1
		P<0.0001
Belgium	NS	rho=+0.96
		P<0.0001
Belize	NS	NS
Bermuda	NS	NS
Bosnia	rho=+0.89	rho=+0.93
	P=0.007	P=0.003
Brazil	rho=+1	NS
	P<0.0001	
British Virgin Islands	NS	NS
Brunei	NS	NS
Bulgaria	rho=+1	rho=+0.96
	P<0.0001	P<0.0001
Canada	NS	NS
Chile	rho=+0.96	NS
	P<0.0001	
Hong Kong	rho=+1	rho=+0.86
	P<0.0001	P=0.014
Costa Rica	rho=+1	rho=+1
	P<0.0001	P<0.0001
Croatia	rho=+1	rho=+1
	P<0.0001	P<0.0001
Cuba	rho=+1	rho=+1
	P<0.0001	P<0.0001
Czech Republic	NS	rho=+0.89
		P=0.007
Denmark	rho=+0.96	rho=+0.89
	P<0.0001	P=0.007
Dominica	NS	NS
Equador	NS	rho=-0.85
		P=0.016
El Salvador	NS	rho=-0.93
		P=0.003
Estonia	NS	rho=+0.86
		P=0.014
Falklands	NS	NS
Finland	NS	NS
France	rho=+0.82	rho=+0.86
	P=0.023	P=0.014
French Guiana	NS	NS
Georgia	rho=+0.96	rho=+0.96
	P<0.0001	P<0.0001
Germany	rho=+0.96	rho=+1
	P<0.0001	P<0.0001
Greece	rho=+0.96	rho=+0.81
	P<0.0001	P=0.027
Guadeloupe	rho=+0.86	NS
	P=0.014	
Guatemala	NS	rho=-0.93
		P=0.003
Guyana	NS	rho=-1
		P<0.0001
Hungary	rho=+0.89	rho=+0.96
	P=0.007	P<0.0001
Iceland	NS	NS
Ireland	rho=-0.96	NS
	P<0.0001	
Israel	rho=+0.96	rho=+1
	P<.0001	P<0.0001
Italy	rho=+1	rho=+1
	P<0.0001	P<0.0001
Jamaica	NS	NS
Japan	NS	rho=+0.96
		P<0.0001
Kazakhstan	NS	NS
Kiribati	rho=-0.87	NS
	P=0.012	
Kuwait	rho=-0.85	rho=-0.78
	P=0.016	P=0.041
Kyrgyzstan	NS	NS
Latvia	NS	rho=+0.89
		P=0.007
Lithuania	NS	rho=+0.89
		P=0.007
Luxenburg	rho=+0.93	rho=+0.86
	P=0.003	P=0.014
Maldives	NS	NS
Malta	NS	NS
Martinique	rho=+0.79	NS
	P=0.039	
Mauritiaus	NS	NS
Mexico	NS	rho=-0.96
		P<0.0001
Monteseraat	NS	NS
Netherlands	NS	NS
New Zealand	NS	rho=-0.96
		P<0.0001
Nicaragua	NS	rho=-0.93
		P=0.003
Norway	NS	NS
Panama	Rho=+0.82	NS
	P=023	
Paraguay	NS	NS
Peru	NS	rho=-0.85
		P=0.016
Poland	NS	NS
Portugal	rho=+1	rho=+1
	P<0.0001	P<0.0001
South Korea	rho=+1	rho=+1
	P<0.0001	P<0.0001
Moldavia	NS	rho=+0.93
		P=0.003
Reunion	NS	NS
Romania	NS	rho=+1
		P<0.0001
Russia	NS	rho=+0.96
		P<0.00 01
St Kitts	NS	NS
St Lucia	NS	NS
St Vincent	NS	NS
Seychelles	NS	NS
Singapore	rho=+1	rho=+1
	P<0.0001	P<0.0001
Slovakia	NS	rho=+0.79
		P=0.036
Slovenia	rho=+1	rho=+0.96
	P<0.0001	P<0.0001
South Africa	NS	NS
Spain	rho=+1	rho=+1
	P<0. 0001	P<0.0001
Surinam	NS	NS
Sweden	rho=+0.96	NS
	P=0.007	
Switzerland	rho=+1	rho=+1
	P<0.0001	P<0.0001
Tajikistan	NS	NS
Macedonia	rho=+1	rho=+1
	P<0.0001	P<0.0001
Trinidad	NS	NS
Turkminstan	NS	NS
Turks and Caico NS NS	NS	NS
Ukraine	rho=+0.79	rho=+1
	P=0.036	P<0.0001
United Kingdom	NS	NS
United States of America	rho=+0.82	NS
	P=0.023	
Venezuela	NS	NS

NS = Not significant. A positive value for rho indicates that the suicide rate increases with increasing age A negative value for rho indicates that the suicide rate decreases with increasing age

A significant increase in suicide rates with ageing was observed in males in 33 countries (including 7 countries where this was the case only for males). A significant increase in suicide rates with increasing ageing was observed in females in 37 countries (including 11 countries where this was the case only for females). These figures included 26 countries with a significant increase in suicide rates with increasing age in both sexes.

There was no significant increase in suicide rates with ageing in males in 59 countries (including 19 countries where this was the case only for males). There was no significant increase in suicide rates with ageing in females in 50 countries (including 10 countries where this was the case only for females). These figures included 40 countries without a significant increase in suicide rates with ageing in both sexes.

Closer examination of countries without an increase in suicide rates with ageing.

Three patterns emerged for countries without a significant increase in suicide rates with ageing in both sexes. First, the suicide rates in a number of these countries was generally low in all the age-bands in both sexes. Second, the suicide rates in a number of these countries generally peaked in the younger age-bands. Third, these countries clustered into three regions: south and central America, the Caribbean and eastern European countries and countries emerging from the former Soviet Union.

Two patterns emerged for countries without a significant increase in suicide rates with ageing in females only. First, the female suicide rates in these countries were generally low. Second, these countries clustered in south and central America.

Two patterns emerged for countries without a significant increase in the suicide rate with ageing in males only. First, there was a peak with the highest suicide rate in the younger age-bands (generally in the age-bands 25-34 years, 35-44 years and 45-54 years) in these countries. Second, these countries clustered in eastern Europe, countries emerging from the former Soviet Union and south and central America. Factors asociated with age-associated trends in suicide rates.

Data on age-associated trends in suicide rates and the GDP, per capita expenditure on healthcare, the proportion of GDP spent on healthcare, life expectancy and child mortality rates were available for 85 countries. Data on age-associated trends in suicide rates and the Gini coefficient were available for 66 countries.

Table 2 illustrates the correlates of age-associated trends in males. In males, there was no significant relationship between age-associated trends in suicide rates and the Gini coefficient. In males, age-associated trends in suicide rates were significantly associated with the GDP (Kruskal-Wallis, X^2^=6.33, 2d.f., P=0.042), per capita expenditure in healthcare (Kruskal-Wallis, X^2^=7.98, 2d.f., P=0.018), the proportion of GDP spent on healthcare (Kruskal-Wallis, X^2^=6.08, 2d.f., P=0.048), life expectancy (Kruskal-Wallis, X^2^=7.4, 2d.f., P=0.024) and child mortality rates (Kruskal-Wallis, X^2^=7.77, 2d.f., P=0.02). There were no differences between countries with a decrease when compared to those with no change or increase in suicide rates with ageing for all these variables. The GDP (Mann Whitney U Test, Z=-2.03, P=0.042), per capita expenditure on healthcare (Mann Whitney U Test, Z=-2.7, P=0.007), the proportion of GDP spent on healthcare (Mann Whitney U Test, Z=-2.5, P=0.012) and life expectancy (Mann Whitney U Test, Z=-2.69, P=0.007) were lower in countries without change when compared to those with an increase in suicide rates with ageing. The child mortality rates were higher in countries without change when compared to those with an increase in suicide rates with ageing (Mann Whitney U Test, Z=2.78, P=0.005).

**Table:** Table 2: **Correlates of age-associated trends in suicide rates in males**

Variable	X2	Degrees of Freedom	Significance
Gini coefficient			NS
GDP	6.33	2	P=0.042
Per capita expenditure on healthcare	7.98	2	P=0.018
Proportion of GDP spent on healthcare	6.08	2	P=0.048
Life expectancy	7.40	2	P=0.024
Child mortality	7.77	2	P=0.02

Kruskal Wallis analysis. NS=Not significant.

Table 3 illustrates the correlates of age-associated in females. In females, there was no significant relationship between age-associated trends in suicide rates and the GDP. In females, age-associated trends in suicide rates were significantly associated with the Gini Coefficient (Kruskal-Wallis, X^2^=12.15, 2d.f., P=0.005), per capita expenditure in healthcare (Kruskal-Wallis, X^2^=10.87, 2d.f., P=0.004), the proportion of GDP spent on healthcare (Kruskal-Wallis, X^2^=6.64, 2d.f., P=0.036), life expectancy (Kruskal-Wallis, X^2^=7.49, 2d.f., P=0.024) and child mortality rates (Kruskal-Wallis, X^2^=16.34, 2d.f., P<0.0001). There were no differences between countries with a decrease when compared to those with no change or increase in suicide rates with ageing for all these variables except per capita expenditure on healthcare, which was lower in countries with a decline when compared to those with no change in suicide rates with ageing (Mann Whitney U Test, Z=-2.4, P=0.017). Per capita expenditure (Mann Whitney U Test, Z=-3.11, P<0.0001), the proportion of GDP spent on healthcare (Mann Whitney U Test, Z=-2.5, P=0.009) and life expectancy (Mann Whitney U Test, Z=-2.4, P=0.015) were lower, and the Gini coefficient (Mann Whitney U Test, Z=-3.29, P<0.0001) and the child mortality rates (Mann Whitney U Test, Z=-3.54, P<0.0001) were higher in countries with a decline when compared to those with an increase in suicide rates with ageing; there were no differences for the GDP. The Gini coefficient (Mann Whitney U Test, Z=-2.17, P=0.03) and the child mortality rates (Mann Whitney U Test, Z=-2.99, P<0.0001) were lower, and life expectancy (Mann Whitney U Test, Z=-2.1, P=0.036) higher in countries without change when compared to those with an increase in suicide rates with ageing; there were no differences for the GDP, per capita expenditure on healthcare and the proportion of GDP spent on healthcare. 

**Table:** Table 3: **Correlates of age-associated trends in suicide rates in females**

Variable	X2	Degrees of Freedom	Significance
Gini coefficient	12.15	2	P=0.005
GDP			NS
Per capita expenditure on healthcare	10.87	2	P=0.004
Proportion of GDP spent on healthcare	6.64	2	P=0.036
Life expectancy	7.49	2	P=0.024
Child mortality	16.34	2	P<0.0001

Kruskal Wallis analysis. NS=Not significant.

## Discussion

Some methodological issues need consideration. Data on suicide rates in cross-national studies should be viewed cautiously.First, data were not available for all countries^22,23^and the validity of this data was unclear.^23,24^Second, the legal criteria for the proof of suicide varies between countries and different regions within a country.^23,25^Third, some countries have poor death registration facilities^25^ andcultural and religious factors and the stigma attached to suicide may lead to under-reporting of suicides.^23,26^However, studies have argued that within individual countries there is internal consistency^27^ and such variation is less important when changes in patterns, rather than the prevalence, of suicide rates is concerned.^28^Moreover, by using an average annual suicide rate calculated from suicide rates for five consecutive years will reduce bias due to year on year random fluctuation in suicide rates.^11^ Comparisons between countries with a decline compared to those with an increase or no change in suicide rates with ageing should be may be subject to Type 2 errors because the number of countries with a decline in suicide rates small, particularly in males.

In countries without an increase in suicide rates with ageing in both sexes or in females only suicide rates were generally low for all the age-bands in both sexes or in females respectively(Table 1). There may have several possible explanations. First, the suicide rate may be low because of under-reporting due to the methodological issues discussed above. Second, the statistical analysis may not have been sufficiently sensitive to detect a correlation between the suicide rate and increasing age due to low suicide rates. Third, the low suicide rates indicate that the absolute number of suicides was also low and, therefore, random variation in the number of suicides in one or two age-bands may inadvertently influence the statistical analysis.^29^However, this was minimized by using an average suicide rate derived from suicide rates for five consecutive years. Fourth, this observed relationship may be genuine. This is supported by similar findings from an earlier cross-national study (2) and within-country studies including: absence of an increase in the suicide rate with increasing age in females in Albania and Finland;^1^ and, in Australia, female suicide rates peaked at menopause and decline thereafter.^14^

In countries without an increase in the suicide rate with increasing age in males, there were peaks with the highest suicide rates in the younger age-bands (Table 1). This observation is consistent with an earlier cross-national study^2^and earlier within-country studies including those from Thailand^8^and the UK.^9,11^Thus, this finding may also be genuine. Moreover, many of the identified age-associated trends in suicide rates in different countries were similar to those observed in an earlier study using older data from the WHO.^2^

Therefore, the observed regional and cross-national differences in the relationship between suicide rates and age needs further consideration because there may be other potential explanations in addition to the methodological issues. The observed lower GDP in countries without a change compared those with an increase in suicide rates with ageing in males (Table 2) is consistent with positive correlations between GDP and general population^30^and elderly^31^suicide rates in within-country and cross-national studies, and a negative correlation between GDP and elderly suicide rates in middle and high income countries.^32,33^The observations of higher Gini coefficient (i.e. greater income inequality) in countries with a decline or without a change compared to those with an increase in suicide rates with ageing in females (Table 3) is also consistent with positive correlation between income inequality and general population^34-36^ and elderly^31,37^suicide rates in within-country and cross-national studies, and identical to findings from an earlier cross-national study of age-associated trends in suicide rates.^21^The findings collectively suggests that suicide rates are less like to increase with ageing in countries with low socio-economic status and greater income inequality. A potential mechanism for this conclusion is examined below.

Societies with low socio-economic status have poorly developed healthcare services.^31,38-41^The amount of expenditure on healthcare is likely to reflect in the degree of development of healthcare services.^31^Per capita expenditure on healthcare and the proportion of GDP spent on healthcare were generally higher in countries with an increase compared to those without a change or a decline in suicide rates with ageing in the current study.

Poorly developed healthcare services may mediate an increase in child mortality rates by being unable to provide primary preventative measures for diseases in childhood (e.g immunisation programmes) and treatment for diseases that are directly related to low socio-economic status (e.g infectious diseases).^31,38,42^This hypothesis is also consistent with higher child mortality rates in countries with lower socio-economic status and greater income inequality^30,31,38^and poorly developed healthcare services.^31,38^Child mortality rates were generally lower in countries with an increase compared to those with no change or a decline in suicide rates with ageing in the current study. This is consistent with similar observation in countries with an increase in suicide rates with ageing in an earlier cross-national study^21^and negative correlations between child mortality rates and general population^30^and elderly^31^suicide rates in cross-national studies.

Increased child mortality rates will, in turn, reduce the life expectancy.^31,38^Given that suicide rates generally increase with age,^1,2^reduced life expectancy will result in fewer people reaching the age of increased risk for suicide in societies with low socio-economic status. This, in turn, will result in a reduced number of elderly suicides in countries with low socio-economic status and greater income inequality. Also, selective survival of those at reduced risk of suicide in old age due to genetic or constitutional factors may further compound this trend.^42^Moreover, in societies with low socio-economic status, those who do survive into old age may be at reduced risk of suicide because they may be able to better tolerate additional hardship in old age due to life-long exposure to adversity,^15,43^and this may also reduce the elderly suicide rate. This has been offered as an explanation for the low suicide rate among elderly African Americans and native Americans in the United States because they often have a life-long history of socio-economic deprivation.^44^The significant positive correlations between elderly suicide rates and the proportion of elderly in the population^40^and elderly dependency ratios^45^also supports this hypothesis because increased life expectancy increases the proportion of elderly in the population. This hypothesis is also consistent with lower life expectancy in countries with lower socio-economic status and greater income inequality,^30,31,38^ poorly developed healthcare services,^31,38^and increased child mortality rates.^30,31,38^ Life expectancy was generally higher in countries with an increase compared to those with no change or a decline in suicide rates with ageing in the current study; this is consistent with similar observation in countries with an increase in suicide rates with ageing in an earlier cross-national study,^21^and positive correlations between life expectancy and general population^30^and elderly^31^suicide rates in cross-national studies.

The above five sequential stages are consistent with those observed in a previous cross-national and cross-sectional study of the relationship between elderly suicide rates and socio-economic status, quality and quantity of healthcare services, child mortality rates and life expectancy.^31^Therefore, the five-sequential stage model proposed in the latter study^31^was adapted to explain the current findings:

(i) presence of a low socio-economic status.

(ii) low socio-economic status leading to poorly developed healthcare services.

(iii) poorly developed healthcare services may interact, mediate and modify other factors and contribute to increased child mortality rates.

(iv) increased child mortality rates leading to reduced life expectancy.

(v) reduced life expectancy leading to fewer people reaching the age of increased risk of suicide in countries with low socio-economic status, and hence absence of an increase in suicide rates with ageing in countries with low socio-economic status.

The observed correlations between age-associated trends in suicide rates and the main variable in each of the five sequential stages are consistent with this model. Caution should be exercised in accepting this five stage sequential model because it has been generated from cross-sectional data using an ecological design. It is, therefore, difficult to be conclusive about the aetiological implications and the findings may merely be an association. Nevertheless, this five stage sequential model appears to be robust because both data from both a cross-national study of elderly suicide rates^31^and age-associated trends in suicide rates (the current study) supported this model. Moreover, these data sets were for different time periods. However, the influence of socio-economic status on age-associated trends may interact with, mediate or modify the effect of other factors not examined in this study. These factors include cross-national differences in rural/urban location, the distribution of protective and risk factors, in the prevalence of mental illness in different age and sex groups,^46^genetic factors that differentially predispose different age and sex groups to mental illness or directly to suicidal behaviour,^46^cultural factors with differential influence on different age and sex groups,^47,48^and cohort effects with differential influence on different age and sex groups.^13,49^
